# Medical school dropouts: regrettable or required?

**DOI:** 10.1080/10872981.2018.1535739

**Published:** 2018-11-08

**Authors:** Husein Rajabali, Rizwan Dewji, Fatemah Dewji

**Affiliations:** Faculty of Medicine, Imperial College London, London, UK

We read the article by Vergel et al. [1] with interest and believe that it raises important points in relation to medical school dropout rates and contributing factors. The article highlighted how through a change in curriculum design, medical school dropout rates had reduced from 41.5% with a traditional curriculum to 3.3%, following a change to an integrated curriculum. Furthermore, it noted an interesting variation in global medical school dropout rates, with a global average of 11.1%, compared to a dropout rate range of 42–63% in South America and developing countries [2].

We recognise the importance of reducing medical school dropout rates, especially in developing countries and South America. The personal, financial and psychological consequences of medical school dropouts cannot be neglected or underestimated. Nonetheless, we believe that it would be short sighted to aim for a global medical school dropout rate of 0%. With the daily challenges and changes in medical practice as well as the increasing requirement for revalidation, tomorrow’s doctors require unparalleled intelligence, perseverance and resilience to overcome the challenges faced as clinical practitioners. As such, it would be an injustice to doctors and patients alike, to modify curriculums and examinations in endeavouring to reduce medical school dropout rates, only to postpone regrettable dropouts of doctors when faced with the unfamiliar and less forgiving challenges of being in medicines front line.

The staggering decrease in dropout rates the article reports raises a key question only touched upon in the article; whether the new curriculum had such impact due to the novel student-centred pedagogic methods and the theorised ‘power shift’ in student–teacher relationships, or whether the syllabus was simply easier. This raises potentially concerning permutations regarding the quality of the graduates. O’Neill et al. [3] note that little research has looked into the effects of medical school curricula on dropout rates, with significance found in areas regarding student attributes such as entry qualifications and socioeconomic status (perhaps indicating why there is such variance in dropout rates globally). However, a Dutch study [4] comparing graduation rates between eight medical schools, running either conventional or active-learning-based curricula, demonstrates an 8% increase when active-learning is employed, indicating that the teaching methods employed could have a far larger impact than previously considered.

Overall, the line of action taken to centre medical education around the learner to reduce dropouts is welcome and required. We feel this system should continue to be implemented in universities and we feel it will not only promote students to remain in medicine but also inspire enthusiasm for the medical sciences and teaching them.

Nonetheless, a level of medical school dropout should be accepted and allowed to ensure the absolute commitment and quality of tomorrow’s doctors.
